# Intrinsic carnosine metabolism in the human kidney

**DOI:** 10.1007/s00726-015-2045-7

**Published:** 2015-07-24

**Authors:** Verena Peters, Celine Q. F. Klessens, Hans J. Baelde, Benjamin Singler, Kimberley A. M. Veraar, Ana Zutinic, Jakub Drozak, Johannes Zschocke, Claus P. Schmitt, Emile de Heer

**Affiliations:** University Children’s Hospital, University of Heidelberg, Im Neuenheimer Feld 672, 69120 Heidelberg, Germany; Department of Pathology, Leiden University Medical Center, Albinusdreef 2, 2333 ZA Leiden, The Netherlands; Department of Metabolic Regulation, Faculty of Biology, University of Warsaw, Krakowskie Przedmieście 26/28, 00-927 Warsaw, Poland; Department of Human Genetics, Medical University of Innsbruck, Innrain 52, Christoph-Probst-Platz, 6020 Innsbruck, Austria

**Keywords:** Carnosine, Anserine, Carnosinase (CNDP1), Metabolism, Diabetic nephropathy

## Abstract

Histidine-containing dipeptides like carnosine and anserine have protective functions in both health and disease. Animal studies suggest that carnosine can be metabolized within the kidney. The goal of this study was to obtain evidence of carnosine metabolism in the human kidney and to provide insight with regards to diabetic nephropathy. Expression, distribution, and localization of carnosinase-1 (CNDP1), carnosine synthase (CARNS), and taurine transporters (TauT) were measured in human kidneys. CNDP1 and CARNS activities were measured in vitro. CNDP1 and CARNS were located primarily in distal and proximal tubules, respectively. Specifically, CNDP1 levels were high in tubular cells and podocytes (20.3 ± 3.4 and 15 ± 3.2 ng/mg, respectively) and considerably lower in endothelial cells (0.5 ± 0.1 ng/mg). CNDP1 expression was correlated with the degradation of carnosine and anserine (*r* = 0.88 and 0.81, respectively). Anserine and carnosine were also detectable by HPLC in the renal cortex. Finally, TauT mRNA and protein were found in all renal epithelial cells. In diabetic patients, CNDP1 seemed to be reallocated to proximal tubules. We report compelling evidence that the kidney has an intrinsic capacity to metabolize carnosine. Both CNDP1 and CARNS are expressed in glomeruli and tubular cells. Carnosine-synthesizing and carnosine-hydrolyzing enzymes are localized in distinct compartments in the nephron and increased CNDP1 levels suggest a higher CNDP1 activity in diabetic kidneys.

**Electronic supplementary material:**

The online version of this article (doi:10.1007/s00726-015-2045-7) contains supplementary material, which is available to authorized users.

## Introduction

Histidine-containing dipeptides such as carnosine (β-alanine-l-histidine) and anserine (β-alanine-l-methyl histidine) are stored in several tissues, with the highest concentrations occurring in skeletal muscle (Bex et al. 2014). These dipeptides have several important protective functions. The best-characterized histidine-containing dipeptide is carnosine (Boldyrev et al. 2013; Budzen and Rymaszewska 2013), which plays many roles in maintaining health, including antioxidant activity (Babizhayev et al. 2013; Boldyrev 1993; Mozdzan et al. 2005) and the ability to scavenge carbonyls (Barski et al. 2013; Negre-Salvayre et al. 2008; Vistoli et al. 2009), inhibit glycation (Alhamdani et al. 2007), and inhibit angiotensin-converting enzymes (Hou et al. 2003; Nakagawa et al. 2006). Carnosine also has several neuroprotective roles (Baek et al. 2014; Boldyrev et al. 2013; Zhang et al. 2011). Anserine has similar benefits, acting as an antioxidant (Kohen et al. 1988) and carbonyl scavenger (Aldini et al. 2005), as well as affecting renal sympathetic nerve activity and blood pressure (Tanida et al. 2010). Functional differences between anserine and carnosine have been reported. For example, anserine has higher anti-radical capacity than carnosine (Boldyrev et al. 2004), lacks anti-crosslinking activity (Hobart et al. 2004), and activates the uptake of calcium by mammalian mitochondria (Daniel et al. 1992).

The naturally occurring amino acid β-alanine is the rate limiting amino acid in the biosynthesis of histidine-containing peptides. β-Alanine is internalized by specific cells in order to synthesize carnosine for intracellular storage. Previous studies found that the taurine transporter (TauT), which is both sodium- and chloride-dependent, is responsible for the uptake of β-alanine in renal cells (Jessen 1994; Jessen and Sheikh 1991).

Carnosine is synthesized by the enzyme carnosine synthase (CARNS), which is present in skeletal and heart muscle, as well as in certain regions in the brain (Veiga-da-Cunha et al. 2014). The gene that encodes CARNS is *ATPGD1* (Drozak et al. 2010); however, the expression and distribution of this enzyme are poorly understood (Boldyrev et al. 2013).

In primates, carnosine is degraded predominantly by the enzyme carnosinase-1 (CNDP1), which is synthesized and secreted by the liver into the circulation; CNDP1 is encoded by the *CNDP1* gene (Teufel et al. 2003). In rodents, CNDP1 is absent in the circulation. CNDP1 is filtered into the urine and reabsorbed into tubular cells, which express CNDP1 within their cytosolic compartment (Teufel et al. 2003). Two forms of carnosinase (CNDP) are expressed in primates: CNDP1, which is also called serum carnosinase, and CNDP2, which is also called tissue carnosinase or cytosolic nonspecific dipeptidase (Teufel et al. 1989).

Given its ability to scavenge reactive oxygen species, carnosine might be beneficial with respect to diabetic nephropathy (DN) (Hipkiss et al. 2001). In animal models with diabetes the renal protective properties of carnosine have been described (Ansurudeen et al. 2012; Peters et al. 2012, 2014; Pfister et al. 2011; Riedl et al. 2011; Yay et al. 2014). With respect to human patients, Janssen et al. (2005) reported that a trinucleotide repeat in the *CNDP1* gene is associated with a differential susceptibility for developing DN in patients with type 2 diabetes. The number of leucine repeats in the leader peptide of the pro-enzyme affects the efficiency of the enzyme secretion (Riedl et al. 2010), thereby altering the effective concentration of this enzyme in the circulation (Mooyaart et al. 2009).

Although the above-mentioned association with microvascular diabetic complications has been supported by several clinical studies, understanding the underlying mechanism requires experimental evidence. Thus, Sauerhofer et al. (2007) generated a transgenic mouse that overexpresses human *CNDP1* under the control of a liver-specific promoter. Giving these mice oral carnosine after induction of diabetes altered their glucose metabolism, but had no significant effect on the development or progression of DN, even though these transgenic mice express human CNDP1 in their serum. These diabetic mice have increased renal CNDP1 activity and reduced renal histidine dipeptide concentrations (Peters et al. 2012), and carnosine supplementation mitigates DN, reduces renal vasculopathy, normalizes vascular permeability (Peters et al. 2012), and improves wound-healing (Ansurudeen et al. 2012). In rats with streptozotocin-induced diabetes, carnosine treatment prevents apoptosis of glomerular cells and podocyte loss (Peters et al. 2014; Riedl et al. 2011), decreases vascular damage (Pfister et al. 2011), and decreases the oxidative damage associated with DN (Yay et al. 2014).

Based on these previously reported findings, we hypothesized that the human kidney is equipped with its own system for metabolizing carnosine. To provide a context for the findings obtained from rodent studies, and to test our hypothesis, we measured the expression level, enzyme activity, distribution, and storage of CNDP1, as well as CARNS, β-alanine uptake levels, and the distribution of TauT in the nephron, in human kidney tissues and in cultured renal cells. We also investigated whether carnosine metabolism differs in DN patients.

## Materials and methods

In this study, we used human kidneys tissue obtained from healthy donors (Eurotransplant); the donor kidneys were unsuitable for transplantation due to technical reasons only; the tissue was de-identified. The organs were collected between 1995 and 2012. The renal cortex and isolated glomeruli were used to investigate the presence of components involved in carnosine metabolism in the kidney and the compartments of the renal cortex.

### Antibodies

To examine the localization of the CNDP1 protein in human tissue samples, we generated a polyclonal anti-CNDP1 antibody. Two rabbits were immunized with a synthetic peptide corresponding to CNDP1, as described by Teufel et al. (2003). The serum was collected and pre-adsorption with the synthetic peptide was used to confirm specificity (Supplementary Fig. 1). The monoclonal anti-CARNS antibody was a generous gift from Prof. Frank L. Margolis (University of Maryland School of Medicine, Baltimore, MD); this antibody has been described previously (Margolis and Grillo 1984; Margolis et al. 1987). The specificity of the anti-CARNS antibody was confirmed by performing double-staining of COS-7 cells transfected with a His-tagged CARNS construct; the antibody showed co-localization with an anti-His antibody (Supplementary Fig. 2). The rabbit anti-TauT antibody (raised against the C-terminal domain of the TauT protein, which is encoded by the *SLC6A6* gene) was obtained from Sigma-Aldrich (St. Louis, MO). For negative controls, the rabbit immunoglobulin fraction (solid-phase absorbed) and normal mouse serum (DakoCytomation, Glostrup, Denmark) were used at the same concentration as their respective primary antibody.

### Immunohistochemistry and immunofluorescence

Immunohistochemistry and immunofluorescence were used to detect the metabolic enzymes CARNS and CNDP1, the localization of histidine-containing dipeptides, and the TauT in the kidney. For CARNS and CNDP1 immunohistochemistry, the tissue sections were deparaffinized, and antigen retrieval was performed by incubating the sections with proteinase K (DakoCytomation) for 10 min at room temperature. Endogenous peroxidases were blocked with 0.125 % H_2_O_2_ (v/v in distilled water) for 20 min. Immunohistochemistry using the anti-TauT antibody was performed as described above except, antigen retrieval was performed using citrate buffer. After antigen retrieval, the sections were incubated for 60 min with primary antibodies against CARNS, CNDP1, or the TauT. After washing with PBS, the sections were incubated with the following secondary antibodies: anti-mouse Envision (DakoCytomation) conjugated with HRP (for anti-CARNS) or anti-rabbit Envision (DakoCytomation) conjugated with HRP (for anti-CNDP1 and anti-TauT). HRP was visualized by incubation with DAB^+^ substrate solution (DakoCytomation) for 10 min. The nuclei were counterstained with hematoxylin.

Double-label immunofluorescence was used to distinguish between the distal and proximal tubules. Tamm-Horsfall was used as a marker of distal tubules. Sections were incubated with anti-CARNS and anti-Tamm-Horsfall and sections were incubated with anti-CARNS and anti-CNDP1 for 60 min. The following secondary antibodies were used: Alexa Fluor 488 donkey anti-goat IgG, Alexa Fluor 546 goat anti-mouse IgG, and Alexa Fluor 488 goat anti-rabbit IgG (all obtained from Life Technologies, Grand Island, NY). As a negative control, the primary antibodies were replaced with normal mouse serum (DakoCytomation) and rabbit immunoglobulin fraction (DakoCytomation) at the same concentration as their respective primary antibody (Supplementary Fig. 3).

### Cultured cells

Cultured cells from various compartments of the kidney were used to examine the cell-specific distribution of carnosine metabolic enzymes. SV40 immortalized human podocytes were used to measure mRNA levels. For measuring protein activity, we used conditionally immortalized mouse podocytes generated from the ImmortoMouse (Charles River, Wilmington, MA) (Weins et al. 2001). Differentiation was induced by growing mouse podocytes ≥10 days on collagen type I (BD Biosciences, Bedford, MA) under permissive conditions at 33 °C with interferon-γ (10 U/ml; Roche Diagnostics, Mannheim, Germany) or under non-permissive conditions at 37 °C without interferon-γ. Podocytes and HK2 tubular epithelial cells (CRL-2190, American Type Culture Collection) were cultured in RPMI 1640 medium (Gibco, Life Technologies, Darmstadt, Germany) supplemented with 10 % FCS (Biochrom GmbH, Berlin, Germany) and penicillin/streptomycin (1 % for HK2 cells and 2 % for podocytes; Biochrom GmbH). Early-passage-number (passage number 8–15) human umbilical vein endothelial cells (HUVEC) were cultured in fetal calf serum containing endothelial cell growth supplement, epidermal growth factor, heparin, hydrocortisone, and 1 % penicillin/streptomycin in accordance with the manufacturer’s instructions (PromoCell GmbH, Heidelberg, Germany). All cells were cultured at 37 °C in 5 % CO_2_ and harvested by adding 60 µl pre-lysis buffer containing 20 mM Tris/HCl (pH 8.0), 150 mM NaCl, 20 mM NaF, 1 % Triton X-100, 2 mM EDTA, 1 mM EGTA (all obtained from Sigma-Aldrich) (Complete Mini, Roche Diagnostics).

### mRNA quantification by RT-PCR

mRNA was isolated from the whole human kidney cortex samples, isolated glomeruli, SV40 immortalized human podocytes, HUVEC cells, and cultured tubular epithelium (HK2) cells, after which *ATPGD1* (which encodes the CARNS protein) and *CNDP1* mRNA levels were quantified using RT-PCR. SYBR Green quantitative PCR was performed to quantify the levels of *ATPGD1* and *CNDP1* mRNA. All cDNA samples were amplified in duplicate. The following primers were used to amplify *ATPGD1* mRNA: forward, GAAGCTGGAGGAGGAGGAG; reverse, GTGGCCTATCACCCTGTGTC. The following primers were used to amplify *CNDP1* mRNA: forward, TTCAATCCGTCTAGTCCCTCACATG; reverse, TGCAATCCACGGGTGTAGTCC. The amplified mRNA levels were normalized to the expression levels of the housekeeping genes *GAPDH* and *HPRT* as described by Baelde et al. (2004).

### Carnosinase protein concentration

CNDP1 protein concentration was measured using a modified ELISA assay (Adelmann et al. 2012). In brief, highly absorbent microtiter plates (Greiner Labortechnik, Frickenhausen, Germany) were coated with 100 µl goat polyclonal anti-human CNDP1 (10 µg/ml; R&D Systems, Wiesbaden, Germany); purified rabbit anti-CNDP1 IgG (Atlas, Abcam, Cambridge, United Kingdom) was used to detect bound CNDP1. A biotinylated goat anti-rabbit IgG was added, followed by avidin-HRP. Deep-blue peroxidase (POD; Roche Diagnostics) was used for color development, and the plates were read immediately at 450 nm. Recombinant human CNDP1 (R&D Systems, Minneapolis, MN) was used as a standard; CNDP1 protein concentrations were measured in the linear part of the dilution curve. The sensitivity of the ELISA assays was approximately 15 ng/ml.

### Anserine and carnosine concentrations

Anserine and carnosine concentrations were measured fluorometrically using high-performance liquid chromatography as previously described (Schönherr 2002). Frozen kidney tissue was homogenized in cold buffer containing 20 mM HEPES, 1 mM ethylene glycol-tetraacetic acid (EGTA), 210 mM mannitol and 70 mM sucrose per gram tissue, pH 7.2. The homogenate was centrifuged at 1500×*g* for 5 min at 4 °C, and the supernatant was kept at −80 °C until analysis. The kidney homogenate and the homogenized cells were diluted with sulfosalicylic acid in order to precipitate the proteins. After the samples were derivatized using carbazole-9-carbonyl chloride, they underwent liquid chromatography and quantification using fluorescence. The retention time of each component was determined by spiking the sample with purified l-carnosine or anserine. All samples were measured at least twice, and one sample was spiked with the standards to identify each analyte. The reliability of the method was 0.91.

### Carnosinase and carnosine synthase activity

CNDP1 activity was assayed as described previously (Peters et al. 2010; Teufel et al. 2003). In brief, the reaction was initiated by the addition of carnosine to cell homogenates at pH 7. The reaction was terminated at pre-determined intervals by adding 1 % trichloroacetic acid. Liberated histidine was derivatized by adding *o*-phthalaldehyde, and fluorescence was read using a MicroTek plate reader (*λ*_Ex_: 360 nm; *λ*_Em_: 460 nm). To avoid nonspecific CNDP2 activity, Bestatin (Sigma-Aldrich, St. Louis, MO) was added to block the activity of CNDP2. Addition of Bestatin did not affect carnosine or anserine degradation, showing that CNDP2 was not active in our experiments. *V*_max_ values were obtained from at least three separate assays by fitting the Dixon plots using a linear regression program. The kinetic parameters were determined using various concentrations of substrates, and the data were fit using the Michaelis–Menten equation.

CARNS activity was determined by measuring the incorporation of radiolabeled β-alanine into carnosine (Drozak et al. 2010). In brief, the reaction was initiated by the addition of [3H]-alanine to cell homogenates. The cell homogenate was then separated by HPLC, and radioactive carnosine was measured using a scintillation counter (Beckman).

### CNDP1 protein in DN patients

For the investigation of the role of carnosine metabolism in relation to renal disease, we used biopsies from patients with type 2 diabetes and DN (*N* = 14) and compared them to healthy controls (*N* = 7) (Baelde et al. 2007). The CNDP1 staining was scored by intensity degrees between 0 and 2.

### Statistical analysis

A minimum of three independent experiments were performed in duplicate. All summary data are provided as mean ± SD. To compare ≥3 groups, a one-way analysis of variance was performed, followed by post hoc analyses using Tukey’s test. For the intensity analysis to compare diabetic patients with controls we used an independent Student *t* test. All statistical analyses were performed using SPSS, version 20.0 (IBM, Armonk, NY).

### Ethical considerations

All tissue samples were coded, then handled and analyzed anonymously in accordance with the ethical principles stated in the Declaration of Helsinki.

## Results

### CNDP1

**Fig. 1:**
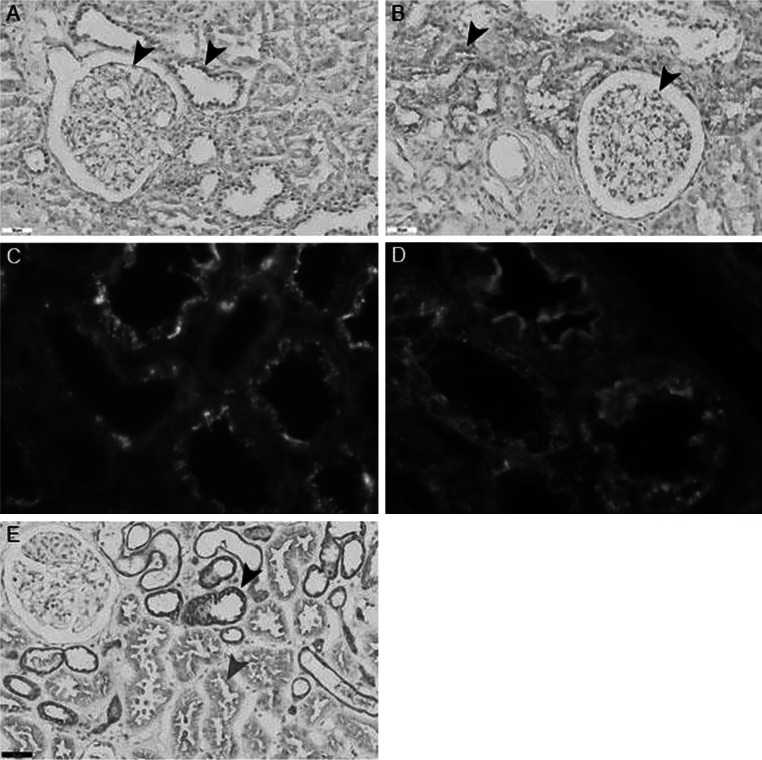
**a** Immunohistochemistry showing the presence of CNDP1 in the glomeruli and distal tubules (*arrows*). **b** Immunohistochemistry showing CARNS expression in proximal tubules and glomeruli (*arrows*). The nuclei were counterstained with hematoxylin. **c** Immunofluorescence showing carnosinase (*red*) and carnosine synthase (*green*) in separate compartments in tubular cells. Immunohistochemistry showing CARNS expression in proximal tubules and glomeruli (*arrows*). **d** Immunofluorescence showing non-overlapping expression of CARNS (*red*) in the proximal tubules and Tamm-Horsfall protein (*green*) in the distal tubules. **e** Immunohistochemistry showing that the TauT is expressed in proximal tubules (*red arrow*) and distal tubules (*black arrow*) in human kidney samples. The highest proteins levels were present in the distal tubules. The nuclei were counterstained with hematoxylin

**Fig. 2:**
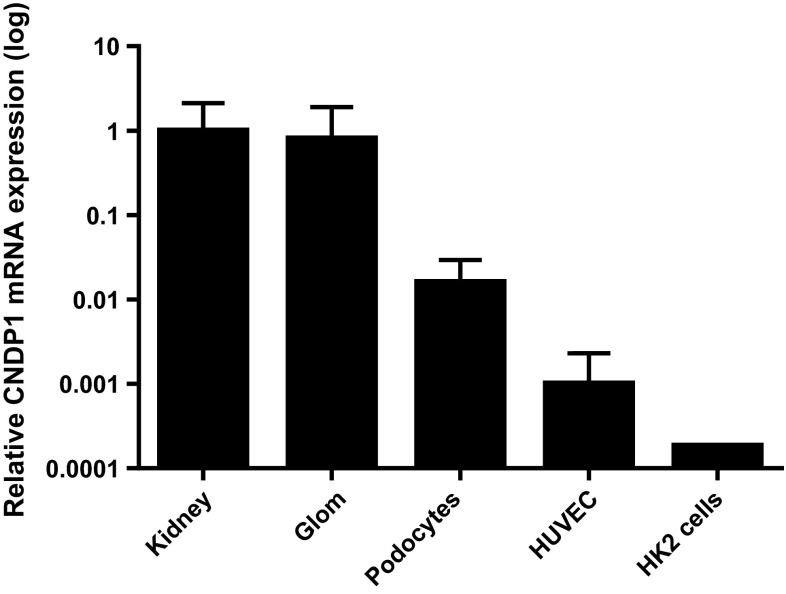
*CDNP1* mRNA was amplified from human kidney samples (1.00 ± 1.12) (*N* = 8), human glomeruli (Glom) (0.802 ± 1.1) (*N* = 8), immortalized human podocytes (0.016 ± 0.0013) (*N* = 2), human endothelial cells (HUVEC) (0.001 ± 0.0013) (*N* = 5), and human proximal tubular epithelial cells (HK2 cells) (0.000185) (*N* = 1). All values were normalized to the mean value obtained from the human kidney samples. Note that the *y*-axis is plotted on a logarithmic scale, expressed as mean ± SD of relative units

**Fig. 3:**
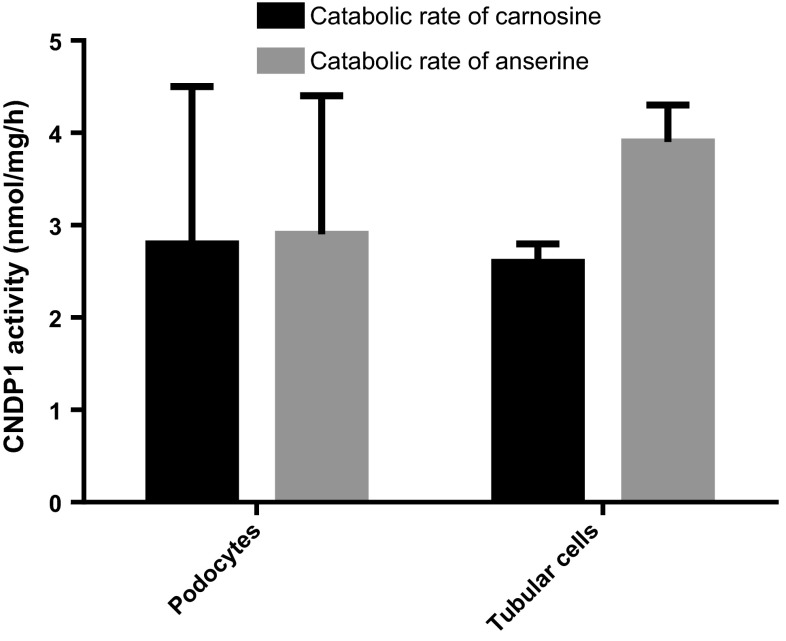
CNDP1 activity (in nmol/mg/h) was measured as the rate of degradation of carnosine (*black*) and anserine (*grey*) in mouse podocytes (2.8 ± 1.7 and 2.9 ± 1.5 nmol/mg/h, respectively) and human proximal tubular epithelial cells (HK2 cells) (1.3 ± 0.4 and 0.05 ± 0.08 nmol/mg/h, respectively), expressed as mean ± SD

Immunohistochemistry showed that the CNDP1 protein is localized primarily in the distal tubules and in the glomeruli (Fig. 1a). Next, we measured the mRNA levels, protein levels, and enzyme activity of CNDP1 in human kidney samples and cultured cells. The relative transcription levels were highest in the human kidney (1.00 ± 1.12 relative units), glomeruli (0.802 ± 1.1), whereas extremely low levels of *CNDP1* mRNA were detected in HUVEC cells (0.001 ± 0.0013) and HK2 cells (0.000185) (Fig. 2). In immortalized podocytes the relative transcription was also high (0.016 ± 0.013). Consistent with this rank order of *CNDP1* expression, CNDP1 protein levels were high in immortalized podocytes (15 ± 3.2 ng/mg protein) and low in HUVEC cells (0.5 ± 0.1 ng/mg protein); interestingly, CNDP1 protein levels were high in HK2 cells (20.3 ± 3.4 ng/mg protein). CNDP1 activity reflected high catabolic rates of carnosine and anserine in podocytes (2.8 ± 1.7 and 2.9 ± 1.5 nmol/mg/h, respectively) and tubular cells (2.6 ± 0.2 and 3.9 ± 0.4 nmol/mg/h, respectively) and low carnosine and anserine catabolic rates in HUVEC cells (1.3 ± 0.4 and 0.05 ± 0.08 nmol/mg/h, respectively) (Fig. 3). Both, CNDP1 protein levels and CNDP1 enzyme activities were correlated to carnosine (*r* = 0.88) and anserine (*r* = 0.81) degradation.

### CARNS

**Fig. 4:**
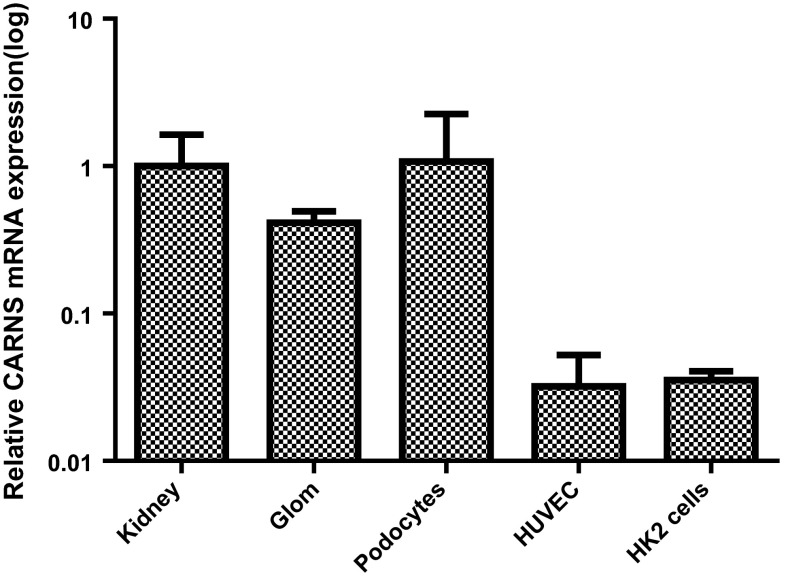
*CARNS* mRNA was amplified from human kidney samples (1.00 ± 0.63) (*N* = 8), glomeruli (Glom) (0.4136 ± 0.08) (*N* = 4), immortalized human podocytes (1.07 ± 0.15) (*N* = 2), human endothelial cells (HUVEC) (0.032 ± 0.02) (*N* = 5), and proximal tubular epithelial cells (HK2 cells) (0.035 ± 0.005) (*N* = 2). All values were normalized to the mean value obtained from the human kidney samples. Note that the *y*-axis is plotted on a logarithmic scale, expressed as mean ± SD of relative units

**Fig. 5:**
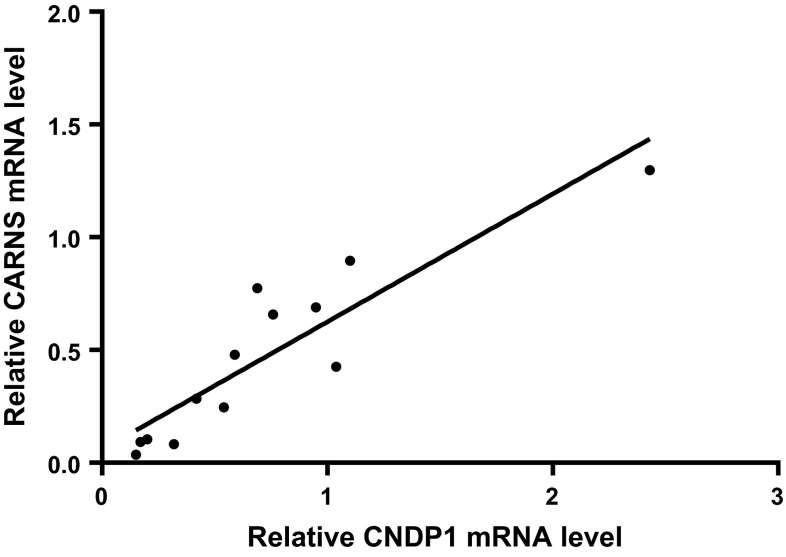
Relative *CARNS* mRNA level is plotted against the relative *CNDP1* mRNA level measured in human kidney samples. Each data point represents a separate sample. The *solid line* is a linear fit of the data. *r* = 0.81 (*N* = 13)

Immunohistochemistry revealed that CARNS protein was primarily localized close to the apical membrane of the proximal tubules, as well as in the glomeruli [albeit at low levels (Fig. 1b)]. Double-label immunofluorescence for CARNS and Tamm-Horsfall protein showed a lack of co-localization, indicating that CARNS is not present in distal tubules. Immunofluorescence revealed a lack of co-localization between CNDP1 and CARNS, as CARNS is localized primarily in proximal tubules (Fig. 1c, d). CARNS mRNA was detected in human kidney samples, (1.00 ± 0.63 relative units), glomeruli (0.4136 ± 0.08), and tubular cells (0.035 ± 0.005) (Fig. 4). CARNS mRNA levels were also high in the immortalized podocyte cell line (1.07 ± 0.15) but were low in the HK2 (0.035 ± 0.005) and HUVEC (0.032 ± 0.02) cells. To examine whether β-alanine affects CARNS activity in the cell lines, HK2 cells were treated with β-alanine. This treatment did not increase CARNS activity in the cells. In normal individual kidney samples, we found relatively high variation of CNDP1 and CARNS levels. Despite this variation, we found a strong positive correlation between *CNDP1* and *CARNS* mRNA levels in individual samples (*r* = 0.81), suggesting that the expression levels of *CNDP1* and *CARNS* are controlled by a similar pathway (Fig. 5).

### Histidine-containing dipeptides

**Fig. 6:**
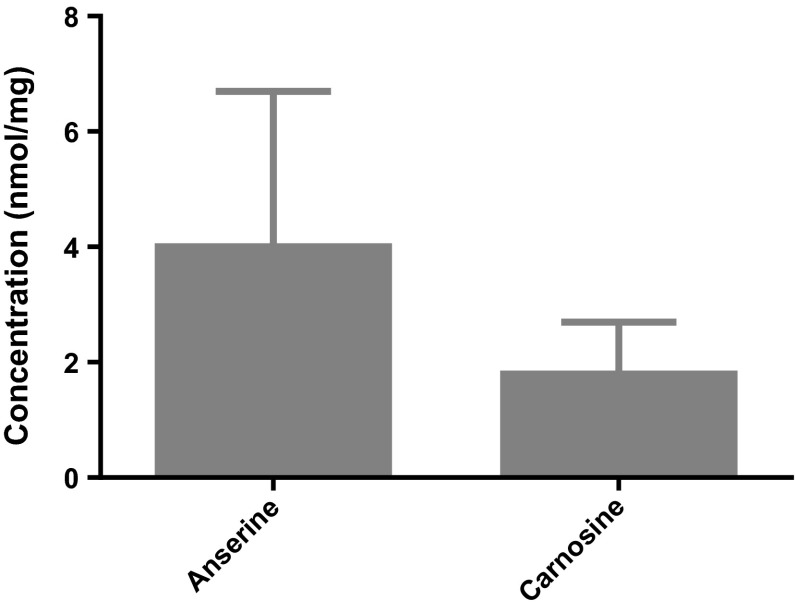
Concentrations of anserine (4 ± 2.7) [95 % CI 2.3–6.4] and carnosine (1.8 ± 0.9) [95 % CI 1.1–2.8] in human kidney samples (*N* = 3) measured using HPLC, expressed as mean ± SD

We next measured the concentrations of histidine-containing dipeptides using high-performance liquid chromatography (HPLC). Repeated measurement of samples coming from three human controls resulted in six values with a mean of 4 nmol/mg anserine and a standard deviation of 2.7 [95 % CI 2.3–6.4] and a mean of 1.8 nmol/mg carnosine and a standard deviation of 0.9 [95 % CI 1.1–2.8] (Fig. 6). Thus, human renal tissue contains more anserine than carnosine. Anserine and carnosine were also present in the cultured podocytes but the concentration varied strongly with concentrations of 2.1–13.8 nmol anserine/mg [95 % CI] and 1.7–8.1 nmol carnosine/mg protein [95 % CI], whereas in tubular cells lower amount of carnosine could be detected (0.2 nmol/mg protein) and anserine was below detection limit.

### Taurine transporter

The TauT transports β-alanine into cells. Immunohistochemistry revealed that the TauT is present in glomerular cells and on the membranes of all renal tubules (Fig. 1e). These data were supported by mRNA measurements (data not shown). Treating HK2 cells dose-dependently (0.1–5 mM) with β-alanine for 24 and 48 h had no effect on the expression of TauT (data not shown).

### CNDP1 protein of DN patients

**Fig. 7:**
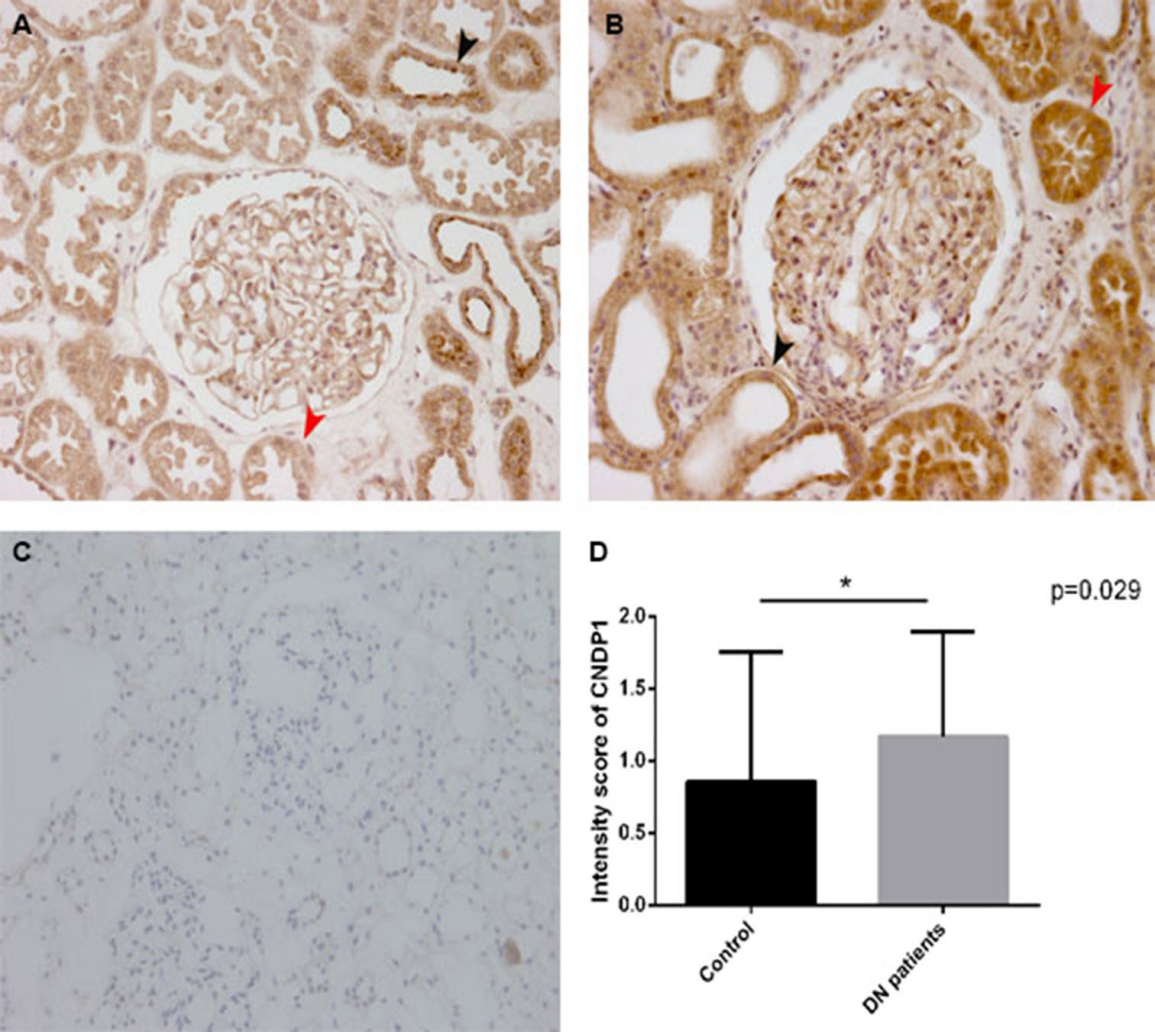
CNDP1 in diabetic patient (*N* = 14) and control (*N* = 7). Immunohistochemistry and intensity score *(*p* = 0.029). It shows a reallocation of CNDP1 from distal to proximal tubules in diabetic patients with renal damage. **a** Healthy control (0.857 ± 0.8997), **b** diabetic nephropathy patients (1.171 ± 0.726), **c** negative control, **d** intensity staining difference; *red arrow* proximal tubules, *black arrow* distal tubules; expressed in mean ± SD

We also compared diabetic patients with DN (*N* = 14) to controls (*N* = 7) by scoring the intensity of CNDP1 immunostaining of renal tissue. In the tubules, we found a significant difference between DN patients and controls (Fig. 7). The higher levels of CNDP1 in DN patients in the proximal tubules could indicate increased hydrolysis of carnosine and anserine. Moreover, CNDP1 might be accumulated in the proximal tubules as a result of reabsorption of CNDP1 caused by proteinuria in patients with DN.

## Discussion

This study provides the first evidence that the human kidney has an intrinsic system for metabolizing carnosine. In addition, we investigated the relation of CNDP1 protein to DN in humans. Combining several experimental approaches, we found that the proteins involved in carnosine metabolism are located in distinct compartments within the nephron. The presence of metabolizing enzymes and the presence of stored histidine-containing dipeptides supports our hypothesis of kidney-specific carnosine metabolism. The staining intensity of CNDP1 was significantly higher in the renal tubules of patients with DN, and immunohistochemistry revealed that CNDP1 was reallocated to the proximal tubules.

We compared the amount of carnosine in the kidney to that in the skeletal muscles fibers, which have the highest concentration of carnosine in the human body. The carnosine concentration determined in human muscles was compared to that in the human kidney. The renal concentrations of anserine (1.1–7.4 mmol/kg for anserine) almost reach the levels of the carnosine concentration of the skeletal muscles (7.2–30.7 mmol/kg dry muscle mass), suggesting that carnosine metabolism plays an important role in maintaining normal kidney function, consistent with the protective properties of carnosine in the muscle. The range of carnosine and anserine concentrations in cultured podocytes differed, and were probably based on the passage number of the cells and culturing conditions.

One of the kidney’s primary functions is to remove and detoxify low-molecular weight compounds. Several studies reported a difference between the protective properties of anserine and carnosine (Boldyrev et al. 2004; Daniel et al. 1992; Hobart et al. 2004). For example, anserine has a higher anti-radical capacity and more antioxidant properties than carnosine; therefore, we hypothesize that anserine protects renal function against the detrimental effects of oxygen radicals.

The podocytes and proximal tubules provide the first line of defense after the fenestrated endothelium. We hypothesize that in these structures, CARNS is required to maintain sufficient anserine concentrations, thereby supporting their protective function. The exchange of protons with K^+^ and Na^+^ ions requires continuous low pH in the distal tubules. Because carnosine has high pH buffering capacity, carnosine must be removed from the distal tubules, thereby explaining the high concentration of CNDP1 in this renal compartment. Teufel et al. (1989) reported high levels of CNDP1 in the stomach epithelium, a site that also requires an extremely low pH.

Next, we found that the TauT is present in the membranes of all renal tubular cells. Taurine and β-alanine compete for binding to TauT (Liu et al. 1992). In the proximal tubules, CARNS synthesizes carnosine from β-alanine and histidine. Therefore, TauT is believed to stimulate the internalization of β-alanine primarily in the proximal tubular epithelium. Because taurine is an osmolyte, the expression of TauT in the distal tubules is regulated by osmolar stress (Chesney et al. 2010) (Fig. 1e). The gene that encodes TauT is regulated by a complex interplay between transcription factors and response elements (Chesney et al. 2010). Over time, β-alanine can deplete taurine from tissues, including renal tissue, thereby upregulating the synthesis and activity of TauT. However, applying β-alanine to HK2 cells did not increase carnosine levels, nor did it appear to induce the expression of either CARNS or the TauT, possibly because the level of TauT protein in the membrane is static.

Although we found clear evidence of organ-specific carnosine metabolism in the kidney and found CNDP1 changes in DN conditions, this study had some limitations. HUVEC cells, podocytes, and HK2 cells were used to investigate the specific locations of the mRNA levels and enzyme activities in the glomeruli. It is possible that when these cell lines were created, they lost part of their original expression profile. Future studies could focus on knockout models with segment specific genetic manipulation.

Considering the physiological function of podocytes in glomerular homeostasis, the high levels of mRNA, proteins, and enzyme activities measured in immortalized podocytes suggest that carnosine metabolism plays a role in glomerular function. In endothelial cells, CNDP1 protein and activity levels were consistently low both in vitro and in vivo; therefore, endothelial cells likely play only a minor role in carnosine metabolism in the kidney. The levels of cell type-specific carnosine and anserine degradation by CNDP1 in renal cells were closely correlated with CNDP1; in other cells, factors such as allosteric conformation and substrate inhibition play a role (Peters et al. 2010, 2011). Interestingly, significant levels of anserine and carnosine were present in the human kidney tissue samples; in contrast, their levels were much lower in the cultured cells, possibly due to low CARNS activity, high CNDP1 activity, intracellular degradation, and/or high turnover.

Our results on DN patients are in line with several studies, which reported that carnosine might play a role in the pathogenesis of DN (Janssen et al. 2005; Sauerhofer et al. 2007). In this respect, diabetic podocyte-specific conditional knockout mice should be developed; these mice would shed light on the role of CNDP1 and CARNS in the glomerulus. Future studies could focus on the regulation of transcription levels, uptake in tubular cells of CNDP1 in DN and investigate whether the histidine-containing dipeptide concentrations are decreased in diabetic state. Also, studies should focus on the role of β-alanine in renal health and disease. In addition, it would be extremely interesting to determine whether diabetes-related oxidative stress changes carnosinase activity in the kidney. In this respect, manipulating renal carnosine metabolism may provide novel therapeutic options for treating DN.

## Electronic supplementary material

Supplementary material 1 (PDF 202 kb)
