# Supplementary material for: Intrinsic carnosine metabolism in the human kidney
Source: Amino Acids. 2015 Jul 24;47(12):2541–50. doi: 10.1007/s00726-015-2045-7 (PMC4633449; doi:10.1007/s00726-015-2045-7)
Supplement: Supplementary file 1 — Supplementary material 1 (PDF 202 kb) [file 726_2015_2045_MOESM1_ESM.pdf]

Supplementary data to Amino Acids

Intrinsic carnosine metabolism in the human kidney

Authors: *Verena Peters[1]*, Celine Klessens[2]*, Hans J. Baelde[2], Benjamin Singler[1], Kimberley Veraar[2], Ana Zutinic[2] , Jakub Drozak[3], Johannes Zschocke[4], Claus P. Schmitt[1], Emile de Heer[2]*
*: These authors contributed equally to this paper

Affiliations: *1:* University Children´s Hospital, University of Heidelberg, Germany*, 2:* Dept. of Pathology, Leiden University Medical Center, The Netherlands, *3*: Dept. of Metabolic Regulation, Faculty of Biology, University of Warsaw, Poland, *4:* Dept. of Human Genetics, Med. Univ. of Innsbruck, Austria

Correspondence:
Celine Q.F. Klessens
Department of Pathology
Leiden University Medical Center, L1Q
Room P0-107
P.O. Box 9600
2300 RC Leiden
The Netherlands
E-mail: c.q.f.klessens@lumc.nl
Telephone: +31(0)71-526 5008
Fax: +31 (0)71-526 6952

Supplementary data

Supplementary figure 1.

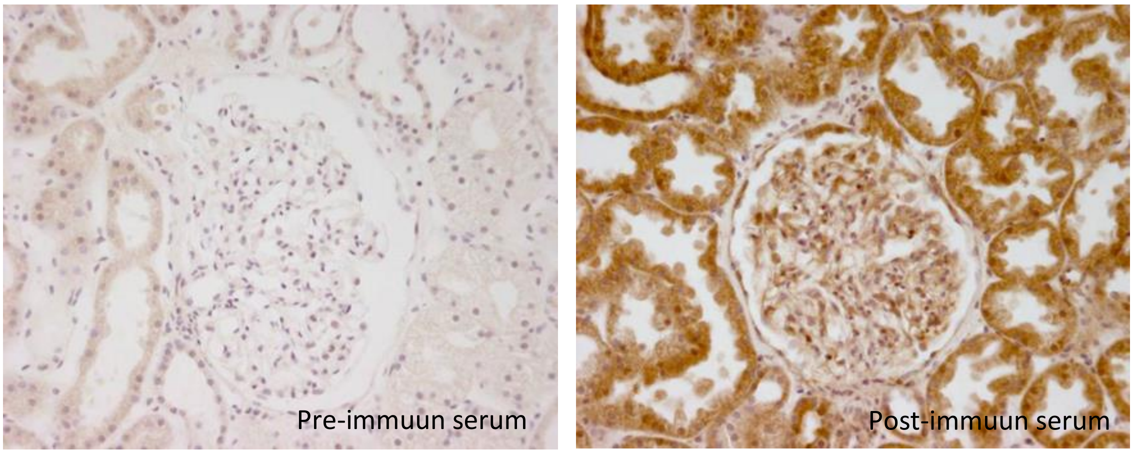

Supplementary figure 1. Carnosinase-1 (CNDP1) antibody specificity
Serum from rabbits immunized by CNDP1 for peptide inhibition. Pre-immuun serum is negative control and post-immuun serum is positive control.

Supplementary figure 2.

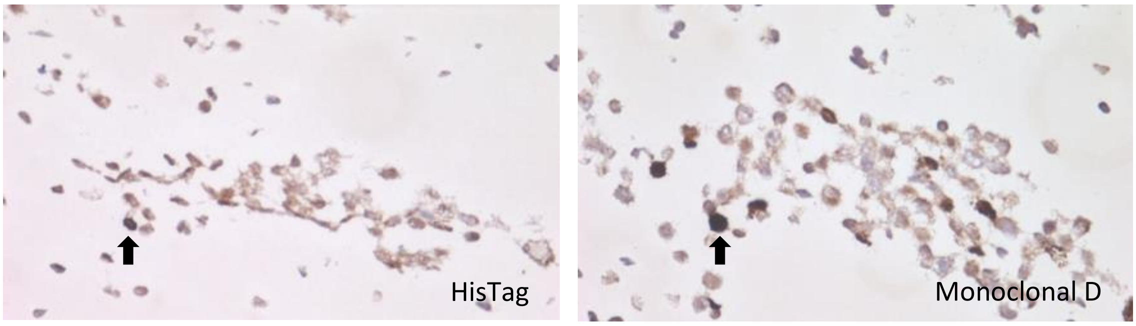

Supplementary figure 2. Carnosine synthase (CARNS) (**ATPGD1) antibody specificity**
The antibody for CARNS was provided by the group of F. Margolis, who isolated the enzyme in a previous study (Margolis, 1987), before the gene for CARNS was discovered in 2010 (Drozak, 2010). By performing double staining with this antibody and anti-HisTag antibody on cryo sections of MNNG cells transfected with HisTag labelled CARNS protein. Arrows show the colocation between the

Supplementary figure 3.

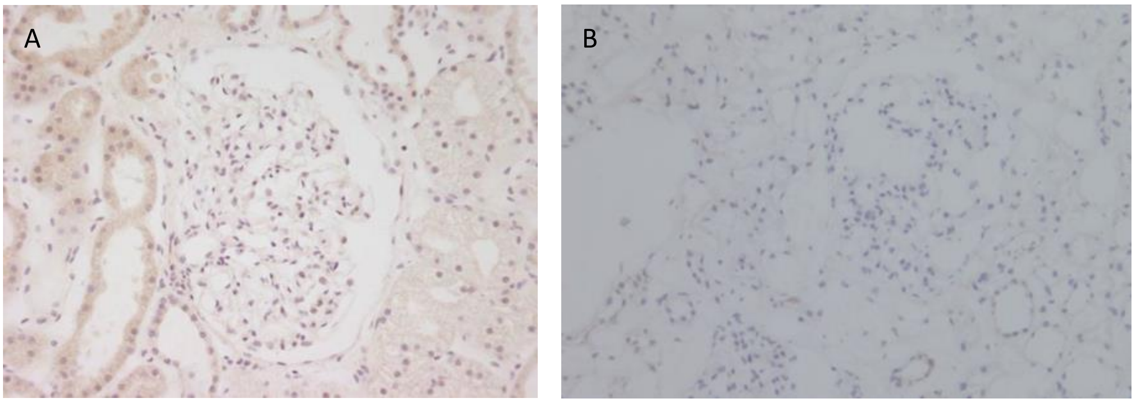

Supplementary figure 3. Negative controls of CNDP1 and CARNS A: negative control of CNDP1(pre-immuun serum)
B: negative control of CARNS (normal mouse serum and immunoglobulin fraction)
